# FGFR2 Might Be a Promising Therapeutic Target for Some Solid Tumors: Analysis of 1312 Cancers with *FGFR2* Abnormalities

**DOI:** 10.3390/ijms262110777

**Published:** 2025-11-05

**Authors:** Hinano Nishikubo, Dongheng Ma, Tomoya Sano, Daiki Imanishi, Takashi Sakuma, Canfeng Fan, Yurie Yamamoto, Motohiro Yamamori, Masakazu Yashiro

**Affiliations:** 1 Molecular Oncology and Therapeutics, Osaka Metropolitan University Graduate School of Medicine, 1-4-3 Asahimachi, Abeno-ku, Osaka 545-8585, Japan; sn23089k@st.omu.ac.jp (H.N.); sg24231c@st.omu.ac.jp (D.M.); sb24524y@st.omu.ac.jp (T.S.); sy23003h@st.omu.ac.jp (D.I.); so22500y@st.omu.ac.jp (T.S.); fancanfeng@gmail.com (C.F.); yurieyamamoto917@gmail.com (Y.Y.); 2 Department of Clinical Pharmacy, School of Pharmacy and Pharmaceutical Sciences, Mukogawa Women’s University, Nishinomiya 663-8179, Japan; moto_y@mukogawa-u.ac.jp; 3 Cancer Center for Translational Research, Osaka Metropolitan University Graduate School of Medicine, 1-4-3 Asahimachi, Abeno-ku, Osaka 545-8585, Japan

**Keywords:** FGFR2, gene alterations, fusion, therapeutic target, solid tumor

## Abstract

Genetic abnormalities of the *fibroblast growth factor receptor 2* (*FGFR2*) gene, including amplification, fusions, and mutations, have been reported in various solid tumors. While molecular targeted therapies against *FGFR2* fusion have been proved to be useful in cholangiocarcinoma, the therapeutic significance of *FGFR2* inhibitors remains unclear in other various solid cancers. Genomic and clinical information from solid tumor cancer gene panel testing cases is consolidated in the Center for Cancer Genomics and Advanced Therapeutics (C-CAT) database in Japan. This study aimed to utilize the C-CAT database to clarify the clinical–pathological significance of *FGFR2* abnormalities. A total of 101,231 patients with solid cancer have been registered in the C-CAT database between June 2019 and June 2025. Of the 101,231 cases, 1312 cases with *FGFR2* gene abnormalities were analyzed. *FGFR2* alterations included amplification in 515 cases, fusion in 280 cases, and mutations in 568 cases. They were detected most frequently in the biliary tract (271 cases), esophagus/stomach (231 cases), and breast (211 cases). Amplification was frequent in the esophagus/stomach (205 cases) and breast (105 cases). Mutations were frequent in the uterus (111 cases), breast (89 cases), and biliary tract (86 cases). Among 515 *FGFR2* alteration cases, *FGFR2* inhibitors were administered in 85 cases. Of the 85 cases, disease control was achieved in 49 cases, 44 cases of which were biliary tract cancer. FGFR2 might be a promising therapeutic target not only for cholangiocarcinoma with fusion but also for esophagus/stomach cancer and breast cancer with *FGFR2* alterations.

## 1. Introduction

*Fibroblast growth factor receptors* (*FGFRs*) are transmembrane receptor tyrosine kinases and consist of four subtypes, i.e., *FGFR-1*, -2, -3, and -*4*. *FGFRs* are involved in a lot of functions such as cell proliferation, differentiation, invasion, and angiogenesis [1,2]. Recently, molecular targeted therapies against the *FGFR2* fusion gene have been approved. Actually, three small molecular inhibitors of FGFR2 including futibatinib [3,4], pemigatinib [5], and tasurgratinib [6] have been approved as of 2024 and are clinically used against cholangiocarcinoma with *FGFR2* fusion genes in Japan. The inhibitors target the ATP binding site of the *FGFR2* kinase domain and inhibit cell proliferation, which suggests that *FGFR2* inhibitors might be effective for cancer with not only *FGFR2* fusion but also active abnormalities [7,8].

Some solid tumors, such as cholangiocarcinoma, endometrial cancer, breast cancer, gastric cancer, and ovarian cancer, have been reported to show *FGFR* abnormalities including amplification, fusion, and mutation. *FGFR2* amplification has been found in 7–23% of breast cancers, 5–10% of gastric cancers, and 12% of endometrial cancers [9,10]. We previously reported that some diffuse types of gastric cancer showed K-samII amplification, which encodes FGFR2 [11], and FGFR2 inhibitors were therapeutically promising for gastric carcinoma with *FGFR2* amplification using several gastric cancer cell lines [12,13,14,15]. Particularly, the extremely malignant type of gastric cancer is associated with *FGFR2* amplification; however, few useful drugs have yet been clinically approved for cancers with *FGFR2* amplification [16]. Furthermore, the therapeutic significance of *FGFR2* abnormalities for these cancers, except cholangiocarcinoma, remains unclear.

Recently, precision medicine has become mainstream for the treatment of tumors based on their genomic abnormalities. Comprehensive genomic panel (CGP) tests have been clinically applied for patients with solid tumors in Japan. Genomic and clinical information of patients who have undergone multi-CGP tests are collected at the Center for Cancer Genomics and Advanced Therapeutics (C-CAT) database in Japan [17,18,19]. A total of 101,231 patients have been registered in the C-CAT database on 30 June 2025. Currently, five types of multi-CGP tests, including FoundationOne CDx (F1), FoundationOne Liquid CDx (F1L), GenMineTOP (GMT), NCC Oncopanel (NCC), and Guardant360 (G360), have been approved in Japan. Analysis using large-scale genomic databases is important to clarify the significance of *FGFR2* abnormalities in carcinomas. The aim of this study is to clarify the clinicopathologic significance of *FGFR2* abnormalities by using the C-CAT database.

## 2. Results

### 2.1. Patient Background of Cases with FGFR2 Gene Abnormalities

Gene abnormalities in the *FGFR2* were detected in 1312 cases out of 101,231 cases by the multi-CGP tests. Table 1 summarizes the patient background of cases with *FGFR2* genetic abnormalities. Among patients with *FGFR2* gene abnormalities, there were 513 males (39.1%) and 799 females (60.9%). More females were detected than males (*p* < 0.001). Furthermore, concerning the age of patients, abnormalities were detected in significantly higher numbers in those under 60 years of age than in those over 60 years of age. Multi-CGP tests more frequently underwent a DNA assay than RNA assay, but there was no significant difference between the two in the detection of the *FGFR2* gene. Submitted specimens were more frequently tissue samples than blood samples, and tissue samples had a significantly higher detection rate of the *FGFR2* gene (*p* < 0.001). Submitted locations of tissue samples were primary and metastasis, and both locations could be detected in cases of *FGFR2* abnormalities. *FGFR2* gene abnormalities were negatively correlated with smoking and tended to be higher in non-drinkers than drinkers.

### 2.2. Distribution of FGFR2 Genetic Abnormalities

**Figure 1:**
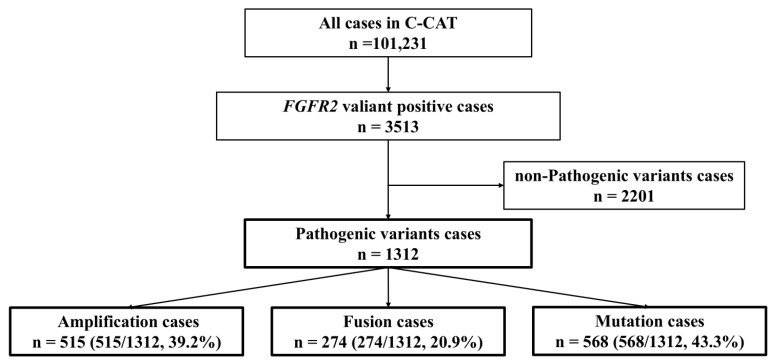
Flow diagram of this study. Among 101,231 cases registered in C-CAT, *FGFR2* gene abnormalities were detected in 3513 cases. Excluding 2201 cases determined to have non-pathogenic variants, 1312 cases were analyzed. Of these 1312 cases, 515 had gene amplification, 274 had fusion genes, and 568 had gene mutations. Among them, 28 cases had both gene amplification and fusion genes coexisting, and 17 cases had both gene amplification and gene mutations coexisting.

In 1312 cancers with *FGFR2* abnormalities, *FGFR2* amplification was detected in 515 cases (39.2%), fusion in 280 cases (20.9%), and mutation in 568 cases (43.3%) (Figure 1).

**Figure 2:**
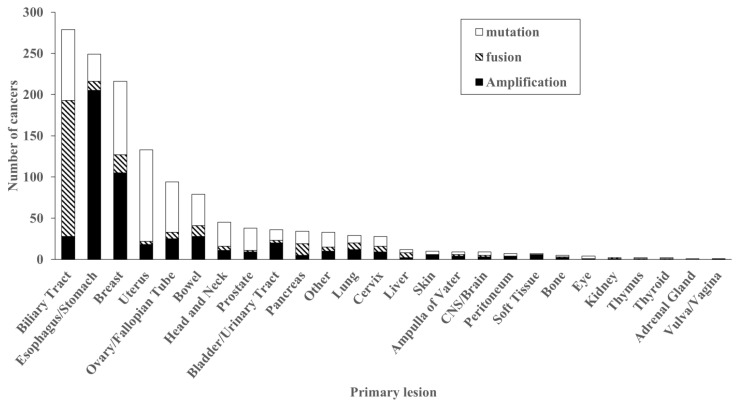
Distribution of *FGFR2* gene abnormalities. Organs in which *FGFR2* gene abnormalities were detected are arranged in descending order. They were most frequently detected in the biliary tract, with fusion genes accounting for half of the cases. Next, they were frequently detected in the esophagus and stomach, where gene amplification accounted for the majority. Organs where gene mutations were frequently detected included the breast, uterus, and ovary/fallopian tube.

Of these, 29 cases showed both gene amplification and fusions, and 16 showed both gene amplification and mutations. Figure 2 summarizes the cancer types with *FGFR2* genetic abnormalities. The most frequent cancer types were found in the biliary tract (271 cases), followed by esophagus/stomach (231 cases), breast (211 cases), uterus (131 cases), and ovary (93 cases).

**Figure 3:**
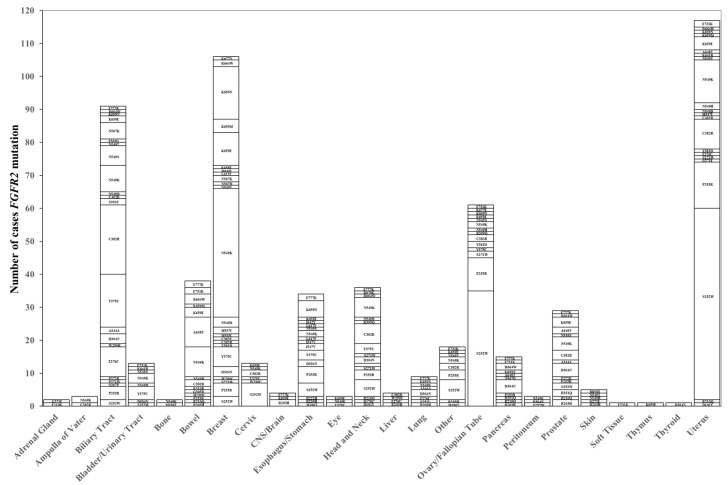
Correlation between tumor types and mutation types of *FGFR2*. A total of 609 cases exhibited *FGFR2* mutation spots. *FGFR2* S252W was a frequent mutation spot (21.8%, 124/568), followed by N549K (16.7%, 95/568). The organ in which *FGFR2* mutation was most frequently detected was the uterus (20.6%, 117/568 cases), followed by breast (18.7%, 106/568 cases).

*FGFR2* amplification was frequently detected in the esophagus/stomach (205 cases), breast (105 cases), biliary tract (28 cases), bowel (28 cases), and ovary/fallopian tube (25 cases). *FGFR2* mutations are frequently detected in the uterus (111 cases), breast (89 cases), biliary tract (86 cases), ovary/fallopian tube (61 cases), and bowel (38 cases). There were frequent hot spot mutations such as S252W (124 cases), N549K (95 cases), C382R (52 cases), P253R (46 cases), and Y375C (35 cases). In contrast, A648T was frequently identified in 23% (9 of 38 cases) of colorectal cancer with *FGFR2* mutations (Figure 3).

*FGFR2* fusions are frequently detected in the biliary tract (163 cases), followed by breast (21 cases), pancreas (13 cases), bowel (12 cases), and esophagus/stomach (11 cases). Some partner genes were frequently identified, such as *BICC1*, *TACC2*, *CTNNA3,* and *KIAA1217* in the biliary tract, while these partner genes were not detected in any other cancers. (Table 2).

### 2.3. Treatment with FGFR Inhibitors

Out of 1312 cancers with the *FGFR2* abnormalities, FGFR2 inhibitors were administered in 85 cases. Of 85 cases, 14 cases received futibatinib, 69 cases received pemigatinib, and 2 cases received both. The cases treated by FGFR2 inhibitors included 77 fusion gene cases, 10 amplification cases, and 2 mutation cases. Of these cases, two cases had both fusion and amplification, and one case had both amplification and mutation. Table 3 shows the correlations between *FGFR2* alteration type and therapeutic response by FGFR2 inhibitors. *FGFR2* gene abnormalities confirmed to be effectively treated with *FGFR2* inhibitors were identified in 44 cases of fusions, 1 case of amplification, 1 case of mutation (N549S), 2 cases of both fusion and amplification, and 1 case of both amplification and mutation.

This summarizes the best response to FGFR2 inhibitor administration and the administered *FGFR2* gene abnormalities. There were 0 cases of CR (complete response), 21 cases of PR (partial response), 27 cases of SD (stable disease), 14 cases of PD (progressive disease), and 23 cases of NE (not evaluable). Most patients who achieved PR received treatment directed at the fusion gene. However, some patients also responded to treatment directed at the gene amplification. In addition, in most cases of SD, treatment was directed at the fusion gene, but disease control was also achieved in some cases where treatment was directed at the gene mutation.

Table 4 shows the correlations between cancer type and therapeutic response by FGFR2 inhibitors. Cancer types were biliary tract (73 cases), esophagus/stomach (3 cases), liver (3 cases), pancreas (2 cases), ampulla of vater (2 cases), and others (2 cases). A total of 48 cases achieved disease control; 44 cases were in the biliary tract, 1 case was in the ampulla of vater, 1 case was in the liver, and there were 2 other cases.

FGFR2 inhibitors were administered to 85 patients, of whom 48 patients (56.5%) achieved disease control (DCR). Most of the DCR cases (44 patients) were in the bile duct, but disease control was also achieved in the ampulla of vater and liver. However, there were 37 cases that did not achieve disease control, including 29 in the bile duct, 3 in the esophagus/stomach, 2 in the liver, 2 in the pancreas, and 1 in the ampulla of vater.

## 3. Discussion

In this study, we analyzed the clinical characteristics of *FGFR2* gene abnormalities and treatment outcomes with inhibitors using a large-scale genomic database in Japan, i.e., the C-CAT database. Our analysis revealed that *FGFR2* gene abnormalities were significantly frequent in women. This finding might suggest that *FGFR2* abnormalities are prevalent in areas such as the uterus, ovaries/fallopian tubes, and breast, as previously reported [20,21].

*FGFR2* gene abnormalities were also significantly frequent in younger patients under 60 years of age. One of the reasons might be that *FGFR2* abnormalities were frequent in cholangiocarcinoma and gastric cancers, which were relatively frequent in younger age [22,23,24]. In addition, *FGFR2* gene abnormalities were negatively correlated with smoking and alcohol. It was indicated that *FGFR2* gene abnormalities may be caused by factors within the tumor without external factors and old ages. No significant differences were found between DNA and RNA analysis in the submitted multi-CGP tests, but the submitted specimens showed significantly higher detection rates in tissue than in liquid.

*FGFR2* amplification was frequent in the esophagus/stomach, followed by the breast and biliary tract, while *FGFR2* fusion was the highest in the biliary tract (for which treatment with *FGFR2* inhibitors is approved in Japan), followed by the breast and pancreas. *FGFR2* mutations were frequently detected in the uterus, breast, and biliary tract. *FGFR2* hotspots such as S252W, P253R, and N549K were frequently detected in endometrial cancer, as previously reported [21,25]. On the other hand, A648T mutation was detected only in colon cancer; this analysis is the first report of this finding.

*FGFR2* fusion partners were several common genes such as *BICC1* and *TACC2*, which are frequently detected in the biliary tract, while *FGFR2* fusion partners of the other cancers were different from these common partner genes. These findings suggest that the mechanisms of FGFR2 fusions may resemble cholangiocarcinoma, but not in the other cancers.

In a recent study, pemigatinib showed no difference in ORR, OS, and PFS between *BICC1* and other partner genes, suggesting that *FGFR2* inhibitors may be effective for cancer with *FGFR2* fusion regardless of the partner gene [5].

The outcome of *FGFR2* inhibitors for *FGFR2* abnormalities revealed that disease control was achieved in 49 of 85 patients. Most of the 49 cases were treated for cholangiocarcinoma. This suggests that *FGFR2* inhibitors were effective for tumors with *FGFR2* fusion. On the other hand, *FGFR2* inhibitors also achieved disease control for not only fusion cases but also gene amplification or mutation. It is necessary to examine the effect of *FGFR2* inhibitors for cancer with *FGFR2* amplification or *FGFR2* mutation.

Currently, the multi-CGP test, Hemesight^®^, has been clinically applied for patients with hematopoietic tumors including leukemia or malignant lymphoma. Hemesight^®^ is also detectable for *FGFR* abnormalities. *FGFR2* inhibitors might be promising for hematopoietic tumors with *FGFR* abnormalities. It will be necessary to examine the significance of *FGFR2* inhibitors for hematopoietic tumors with *FGFR* abnormalities using C-CAT data in the near future.

## 4. Materials and Methods

### 4.1. Extraction of Subject

This study used a total of 101,231 cases registered in the C-CAT database from June 2019 to June 2025. Only facilities authorized to use C-CAT can access this information through the data sharing system of the C-CAT Research Use Portal (https://www.ncc.go.jp/en/c_cat/use/index.html (accessed on 19 September 2025).

Of these data, 3512 cases had *FGFR2* gene abnormalities. The gene alteration of a tumor was examined by one of five multi-CGP tests: FoundationOne^®^ CDx (F1; Foundation Medicine Inc., Cambridge, MA, USA), OncoGuide™ NCC Oncopanel System (NCC; Sysmex Co., Ltd., Kobe, Japan), GenMineTOP^®^ Cancer Genome Profiling Systems (GMT; KONICA MI-NOLTA REALM Co., Inc., Tokyo, Japan), FoundationOne^®^ Liquid CDx (F1L; Foundation Medicine Inc.), or Guardant360^®^ CDx sequencing technology (G360; Guardant Health, Palo Alto, CA, USA). F1 was performed using genomic DNA extracted from tumor tissue. The NCC test analyzed both tumor tissue and circulating tumor DNA (ctDNA) in the blood, while the GMT test used DNA and RNA extracted from tumor tissue as well as DNA from whole blood samples. F1L and G360 tests, on the other hand, use peripheral blood to examine tumor-derived ctDNA. Regarding the number of genes analyzed, the NCC test examined 114 gene mutations [26], the F1 test examined 324 gene mutations [27], and the F1L test, which analyzes circulating ctDNA in the blood, also examined 324 gene mutations [28]. The GMT test, on the other hand, examined a total of 737 gene mutations [29]. All five tests were capable of detecting FGFR2 gene abnormalities. Of these, F1 was used in 69,275 cases, F1L was used in 15,445 cases, GMT was used in 4737 cases, NCC was used in 9473 cases, and G360 was used in 2301 cases. This study was approved by the C-CAT review committee (C-CAT management number: CDU2022-044N) and by the medical ethics committee of Osaka Metropolitan University (approval number 2022-111).

### 4.2. Extraction of Genetic Abnormalities

Multi-CGP tests use next-generation sequencers (NGSs) to detect nucleotide substitutions, insertion/deletion, amplifications/deletions, and fusions. Among the genes extracted from cancer gene panel tests, only those evaluated as pathogenic, likely pathogenic, oncogenic, or likely oncogenic in the clinical annotation of C-CAT findings were extracted. In this study, genes were selected to evaluate the true targets, and variants of uncertain significance (VUS) were not included. Pathogenic, likely pathogenic, oncogenic, likely oncogenic, and VUS were evaluated based on the definitions outlined in the interpretation guidelines for sequence variants established by a joint consensus recommendation from the American College of Medical Genetics and Genomics and the Association for Molecular Pathology. Oncogenic/likely oncogenic classifications follow C-CAT’s proprietary clinical significance criteria. These classifications serve as evidence demonstrating the associations between genetic abnormalities and cancer, based on database analysis and research findings.

### 4.3. Patients’ Clinicopathologic Analysis

A retrospective study was conducted using the C-CAT database, which includes clinicopathologic information such as age, gender, smoking, alcohol, assay sample, sample type, and sampling site, cancer type, genomic abnormalities such as mutation, amplification, and fusions, and best response. Treatment efficacy was assessed as complete response (CR), partial response (PR), stable disease (SD), progressive disease (PD), or not evaluable (NE) based on the best response assessment for patients treated with FGFR2 inhibitors. The overall response rate (ORR) was defined as CR or PR, and the disease control rate (DCR) was defined as CR, PR, or SD.

### 4.4. Statistical Analysis

Significance tests were conducted using the chi-square test or Fisher’s exact probability test. A *p*-value of <0.05 was defined as statistically significant in all tests. Statistical analyses were performed using SPSS^®^ version 28 (IBM Corp., Armonk, NY, USA) and the EZR (Easy R) software package version 1.65 (Saitama Medical Center, Jichi Medical University, Saitama, Japan).

## 5. Conclusions

This study revealed the distribution of *FGFR2* abnormalities in some types of carcinomas and suggested the clinical usefulness of *FGFR2* inhibitors for tumors with *FGFR2* abnormalities not only fusion but also amplification and mutation.

## Figures and Tables

**Table 1:** Correlations between *FGFR2* alteration and samples.

Patient and Samples		*FGFR2* Alteration (n = 1312)	*FGFR2* Wild (n = 99,919)	*p*-Value
Gender	Male (n = 50,437)	513 (39.1%)	49,924 (50.0%)	
	Female (n = 50,790)	799 (60.9%)	49,991 (50.0%)	<0.001
Age	<60 (n = 38,129)	539 (41.1%)	37,406 (37.4%)	
	60 ≦ (n = 63,103)	773 (58.9%)	62,514 (62.6%)	<0.01
Smoking	Positive (n = 43,945)	476 (36.3%)	43,469 (43.5%)	
	Negative (n = 51,599)	763 (58.2%)	50,836 (50.9%)	<0.001
Alcohol	Positive (n = 11,627)	138 (10.5%)	11,489 (11.5%)	
	Negative (n = 77,339)	1031 (78.6%)	76,308 (76.4%)	<0.001
Assay sample	DNA assay (n = 96,494)	1261 (96.1%)	95,233 (95.3%)	
	RNA assay (n = 4737)	51 (3.9%)	4686 (4.7%)	N.S.
Sample type	Tissue (n = 83,485)	1126 (85.8%)	82,359 (82.4%)	
	Blood (n = 17,746)	186 (14.2%)	17,560 (17.6%)	<0.001
Sampling site	Primary tumor (n = 57,323)	797 (60.7%)	56,526 (56.6%)	
	Metastatic tumor (n = 25,777)	326 (24.8%)	25,451 (25.5%)	<0.001

When comparing patient backgrounds between *FGFR2* alteration and *FGFR2* wild, significant differences were observed in sex, age, smoking, alcohol consumption, submitted specimens, and the site of specimen collection (*p* < 0.01).

**Table 2:** Fusion partners of *FGFR2* in each organ.

Organ	Fusion Partner (Number of Cases)
Ampulla of Vater (n = 2)	*CCSER2* (1), *RBM2* (1)
Biliary Tract (n = 165)	*BICC1* (29), *TACC2* (9), *AHCYL1* (6), *CCDC6* (5), *VCL* (5), *CTNNA3* (6), *KIAA1217* (6), *PAWR* (5), *KIAA1598* (4), *SHROOM3* (4), *INA* (3), *TACC1* (3), *AFF3* (2), *ANKRD28* (2), *BEND3* (2), *CASP7* (2), *CCAR1* (2), *CLIP1* (2), *COL16A1* (2), *DDX21* (2), *NOL4* (2), *NRBF2* (2), *POC1B* (2), *RBFOX2* (2), *SORBS1* (2), *SORBS2* (2), *SYNPO2* (2), *A1CF* (1), *ALS2CR12* (1), *ARHGAP21* (1), *ATAD2* (1), *BAIAP2L1* (1), *CBY1* (1), *CCDC147* (1), *CCDC171* (1), *CCSER2* (1), *CCT7* (1), *CD2AP* (1), *CLASP2* (1), *CROCC* (1), *CUX1* (1), *DNAH8* (1), *DSP* (1), *EBF3* (1), *EEA1* (1), *FOXP1* (1), *GPHN* (1), *GPX3* (1), *GRB2* (1), *HDX* (1), *HOOK1* (1), *KIAA1210* (1), *MITF* (1), *MYLK* (1), *NELL2* (1), *NRAP* (1), *PAH* (1), *PDE4DIP* (1), *PKD2L1* (1), *POLDIP3* (1), *PPP1R21* (1), *PRDM16* (1), *RASAL2* (1), *RMND1* (1), *SEC63* (1), *14-Sep* (1), *SLMAP* (1), *TRA2B* (1), *TRIM54* (1), *TTC28* (1), *TULP3* (1), *TXLNA* (1), *UBE2K* (1), *WAC* (1), *WARS1* (1), *WDR65* (1), *ZMYM4* (1)
Bladder/Urinary Tract (n = 3)	*TACC2* (2), *CCDC6* (1)
Bone (n = 1)	*ATE1* (1)
Bowel (n = 12)	*TACC2* (2), *ATE1* (1), *CIT* (1), *FOXP1* (1), *GLS* (1), *KIAA1217* (1), *POC1B* (1), *POF1B* (1), *PTBP3* (1), *STK31* (1), *TBC1D1* (1)
Breast (n = 22)	*ATE1* (3), *C10orf88* (1), *CCDC6* (1), *CCSER2* (1), *CLIP1* (1), *CPXM2* (1), *CTNNA3* (1), *FAM160B1* (1), *GAB2* (1), *KIAA1598* (1), *KIF11* (1), *MPP7* (1), *PRDX3* (1), *PTPRE* (1), *RBM20* (1), *RGS10* (1), *SLMAP* (1), *TACC2* (1), *VCL* (1)
Cervix (n = 7)	*TACC2* (2), *ATE1* (1), *BICC1* (1), *CASP7* (1), *FARS2* (1), *PRKN* (1)
CNS/Brain (n = 2)	*SOGA1* (1), *UGP2* (1)
Esophagus/Stomach (n = 11)	*TACC2* (3), *TRIM44* (2), *ATE1* (2), *DMBT1* (1), *LAMP1* (1), *PPAPDC1A* (1), *SKIL* (1)
Head and Neck (n = 5)	*CCDC102A* (1), *FILIP1* (1), *FOXP1* (1), *KIAA1217* (1), *PBLD* (1)
Kidney (n = 1)	*KIFC1* (1)
Liver (n = 6)	*BICC1* (3), *AMTN* (1), *CCDC6* (1), *KIAA1217* (1)
Lung (n = 8)	*CIT* (1), *CTNNA3* (1), *EEA1* (1), *FOXP4* (1), *MCC* (1), *NAV2* (1), *NRBF2* (1), *TACC2* (1)
Other (n = 5)	*BICC1* (2), *ACPP* (1), *CCDC6* (1), *YPEL5* (1)
Ovary/Fallopian Tube (n = 8)	*C10orf11* (1), *DLG5* (1), *KIAA1217* (1), *KIF24* (1), *RASEF* (1), *TRIM23* (1), *TRIM8* (1), *ZMYND11* (1)
Pancreas (n = 14)	*SYCP1* (2), *CAT* (1), *CGNL1* (1), *CIT* (1), *FOXP1* (1), *FRMD4A* (1), *KIAA1598* (1), *POLDIP3* (1), *TACC2* (1), *TBC1D1* (1), *TFCP2* (1), *TMEM132B* (1), *TXLNA* (1)
Prostate (n = 2)	*CTNNA3* (1), *TACC2* (1)
Thymus (n = 1)	*KIAA1217* (1)
Thyroid (n = 1)	*TACC2* (1)
Uterus (n = 4)	*ACTN1* (1), *ATE1* (1), *CCDC6* (1), *PPP2R5C* (1)

A total of 274 cases exhibited *FGFR2* fusion genes. *BICC1* was a frequent fusion partner (12.8%, 35/274). The organ in which *FGFR2* fusion was most frequently detected was the biliary tract (60.2%, 165/274 cases), followed by breast (8.0%, 22/274 cases).

**Table 3:** Correlations between *FGFR2* alteration type and therapeutic response by FGFR2 inhibitors.

Therapeutic Response of FGFR2 Inhibitors	*FGFR2* Alteration Type				
	Amplification (n = 8)	Fusion (n = 138)	Mutation (n = 2)	Amplification and Fusion (n = 4)	Mutation and Fusion (n = 2)
CR (n = 0)	0	0	0	0	0
PR (n = 21)	1	20	0	0	0
SD (n = 27)	0	24	1	1	1
PD (n = 14)	1	12	0	1	0
NE (n = 23)	4	18	0	1	0
ORR; CR + PR (n = 21)	1	20	0	0	0
DCR; CR + PR + SD (n = 48)	1	44	1	1	1

CR, complete response; PR, partial response; SD, stable disease; PD, progressive disease; NE, not evaluable; ORR, overall response rate; DCR, disease control rate.

**Table 4:** Correlations between cancer type and therapeutic response by FGFR2 inhibitors.

Cancer Type	DCR (n = 48)	PD + NE (n = 37)
Ampulla of vater (n = 2)	1	1
Biliary tract (n = 73)	44	29
Esophagus/Stomach (n = 3)	0	3
Liver (n = 3)	1	2
Other (n = 2)	2	0
Pancreas (n = 2)	0	2

CR, complete response; PR, partial response; SD, stable disease; PD, progressive disease; NE, not evaluable; DCR, disease control rate: CR + PR + SD.

## Data Availability

The datasets presented in this article are not readily available because the data are part of an ongoing study or due to limitations. Requests to access the datasets should be directed to the Center for Cancer Genomics and Advanced Therapeutics (C-CAT).
