# Correction: Nishikubo et al. FGFR2 Might Be a Promising Therapeutic Target for Some Solid Tumors: Analysis of 1312 Cancers with *FGFR2* Abnormalities. *Int. J. Mol. Sci.* 2025, *26*, 10777

**DOI:** 10.3390/ijms27020665

**Published:** 2026-01-09

**Authors:** Hinano Nishikubo, Dongheng Ma, Tomoya Sano, Daiki Imanishi, Takashi Sakuma, Canfeng Fan, Yurie Yamamoto, Motohiro Yamamori, Masakazu Yashiro

**Affiliations:** 1 Molecular Oncology and Therapeutics, Osaka Metropolitan University Graduate School of Medicine, 1-4-3 Asahimachi, Abeno-ku, Osaka 545-8585, Japan; sn23089k@st.omu.ac.jp (H.N.); sg24231c@st.omu.ac.jp (D.M.); sb24524y@st.omu.ac.jp (T.S.); sy23003h@st.omu.ac.jp (D.I.); so22500y@st.omu.ac.jp (T.S.); fancanfeng@gmail.com (C.F.); yurieyamamoto917@gmail.com (Y.Y.); 2 Department of Clinical Pharmacy, School of Pharmacy and Pharmaceutical Sciences, Mukogawa Women’s University, Nishinomiya 663-8179, Japan; moto_y@mukogawa-u.ac.jp; 3 Cancer Center for Translational Research, Osaka Metropolitan University Graduate School of Medicine, 1-4-3 Asahimachi, Abeno-ku, Osaka 545-8585, Japan

In the original publication [1], the Institutional Review Board Statement and Informed Consent Statement were not included. In addition, the authors modified the Abstract after identifying an error; the phrase “Among 515 *FGFR2* alteration cases” to “Among 1312 *FGFR2* alteration cases”. The correct Institutional Review Board Statement and Informed Consent Statement appear below. The authors state that the scientific conclusions are unaffected. This correction was approved by the Academic Editor. The original publication has also been updated.

**Institutional Review Board Statement:** This study was approved by the C-CAT review committee (C-CAT management number: CDU2022-044N on 29 November 2022) and by the medical ethics committee of Osaka Metropolitan University (approval number 2022-111 on 15 September 2022).
**Informed Consent Statement:** Informed consent was waived by the Medical Ethics Committee of Osaka Metropolitan University.
